# Access to Chronic Pain Services for Adults from Minority Ethnic Groups in the United Kingdom (UK): a Scoping Review

**DOI:** 10.1007/s40615-023-01803-2

**Published:** 2023-10-16

**Authors:** Emily Leach, Mwidimi Ndosi, Gareth T. Jones, Helen Ambler, Sophie Park, Jennifer S. Lewis

**Affiliations:** 1 School of Health and Social Wellbeing, University of the West of England, Bristol, UK; 2 Solent NHS Podiatry, Solent NHS Trust, Southampton, UK; 3 School of Medicine, Medical Sciences and Nutrition, University of Aberdeen, Aberdeen, UK; 4 Bath National Pain Centre, Royal United Hospitals Bath NHS Trust, Bath, UK

**Keywords:** Ethnicity, Chronic pain, Pain management, Adults, United Kingdom, Scoping review

## Abstract

**Background:**

Chronic pain services in the UK are required to provide services which meet the diverse needs of patients, but little is known about the access and use of these services by minority ethnic groups.

**Objective:**

To assess the available evidence regarding the ethnic profile of adults who access secondary and tertiary chronic pain services in the UK.

**Methods:**

A scoping review was conducted (August 2021–October 2021), comprising comprehensive literature searches using Embase, Medline and CINAHL databases and the grey literature. Studies were included if they reported on (i) access to chronic pain services in secondary and/or tertiary care in the UK, (ii) adults and (iii) stated the ethnicity of the involved participants. Studies were included if published between 2004 and 2021, as demographic data during this period would be broadly representative of the UK population, as per the 2021 UK census. A descriptive synthesis of the extracted data was performed.

**Results:**

The search yielded 124 records after duplicates were removed. Following title and abstract screening, 44 full texts were screened, ten of which were included in the review.

**Conclusions:**

This is the first review to explore access to chronic pain services for adults from minority ethnic groups in the UK. Given the limited number of studies that met the inclusion criteria, the review highlights the need for routine collection of ethnicity data using consistent ethnic categories within UK chronic pain services and increased involvement of minority ethnic groups within chronic pain research. Findings should inform future research that aims to improve access to UK chronic pain services for adults from minority ethnic groups.

**Supplementary Information:**

The online version contains supplementary material available at 10.1007/s40615-023-01803-2.

## Introduction

Chronic pain is a considerable healthcare concern, particularly for adults in the UK. Chronic pain is estimated to affect between 35-51.3% of adults in the UK population [1] compared to 11–38% of children and adolescents [2]. However, the prevalence of chronic pain amongst adults from minority ethnic groups in the UK is unclear, and debate within the literature exists. Some studies indicate the prevalence of chronic pain is similar between adults from different ethnic groups [3], whereas others suggest a higher prevalence of chronic pain in adults from minority ethnic groups [4, 5].

Access to a chronic pain service is recognised as an important aspect of treatment [6, 7]. Yet, there are shortfalls in the delivery of pain services across the UK in terms of structure and specialist staffing [8, 9]. It is estimated that one pain treatment facility exists for up to every 370,000 people living in the UK [10]. This number appears insufficient given that up to 35,000,000 adults from the current UK population experience chronic pain [11]. This suggests there is inadequate provision of these services across the whole population regardless of ethnicity.

The National Health Service (NHS) quality and diversity guidance states that all services, including chronic pain services, should support equality and deliver services which meet the varied needs of patients, including those from minority ethnic groups [12]. Ensuring minority ethnic groups have access to services and improved health outcomes has been identified as an NHS priority given that race is a recognised protected characteristic [13]. Yet, it has been speculated that there is poorer access to chronic pain services across the UK for non-white ethnic groups compared to white ethnic groups [8].

While there is clear evidence of ethnic disparities in the treatment of adults from minority ethnic groups with chronic pain [14–16], less attention has been paid to issues of accessing these services. Given the NHS’s prioritisation of improved access to services [9], it is crucial to understand the current population of adults in the UK with chronic pain that are accessing chronic pain services in secondary and/or tertiary care.

Chronic pain prevalence, perceptions and outcomes of minority ethnic groups compared to white populations have been assessed in the literature [13, 17, 18]. However, the majority of this research has been undertaken outside of the UK. Little is known about the access to and uptake of chronic pain services for minority ethnic groups within the UK. This scoping review aims to address this gap in knowledge.

The objective of this study was to examine the existing literature to determine the extent to which adults with chronic pain from minority ethnic groups access secondary and/or tertiary chronic pain services in the UK and to identify further research related to ethnic disparities within secondary and tertiary chronic pain services in the UK.

## Methods

### Design

We conducted a scoping review exploring the body of literature relating to access to chronic pain services for adults from minority ethnic groups in the UK given that this has not yet been comprehensively reviewed. Our intention was therefore to draw broad conclusions of the cumulative evidence on this topic [19, 20]. We define minority ethnic groups as all ethnic groups except the white British group as per UK Government recommendations [21]. The protocol for this review is published elsewhere as a peer-reviewed article [22], so a summary of methods will be described here.

### Information Sources

A systematic search of Medline, Embase and CINAHL bibliographic databases was conducted between August 2021 and October 2021. A search in Google Scholar and Open Grey for grey literature including clinical guidelines, policy documents and reports was also conducted. Contact with experts in pain management was sought to obtain additional resources.

### Search Strategy

The PICO model was used to formulate the search strategy by identifying key concepts (Supplementary Material Table 1). The outlined bibliographic databases were searched electronically using combinations of keywords and indexed search terms (Supplementary Material Table 2). Reference lists of included articles were checked to identify any potentially eligible studies and to reduce the risk of missing preliminary evidence [31].

### Study Selection

The inclusion and exclusion criteria outlined in Table 1 were used in the screening of records and assessing for eligibility.

**Table 1:** Inclusion and exclusion criteria

*Papers were included if they met any of the following:*	*Papers were excluded if they met any of the following:*
Report on access to pain treatment settings (including pain management clinics, pain clinics, pain programmes, pain management services, pain management programmes) in secondary and/or tertiary care in the UK.	Research conducted in primary care only. Any non-UK-based study
Adult population with chronic pain and state the ethnicity of the involved participants within the demographics.	Paediatric population, i.e. under 18 years of age.
Primary research studies including observational studies with cross-sectional or prospective research design, case-control studies, systematic reviews and studies with experimental designs, qualitative interviews and case studies. Published abstracts and grey literature sources were also included.	Acute or malignant pain.
Publication between 2004 and 2021 as this time period acknowledges the joining of eight Central and Eastern European countries to the European Union in 2004 and should represent the current UK population recorded in the 2021 UK census.	

### Study Screening

Once duplications were removed, the study titles and abstracts were initially screened by the lead and senior authors. Studies selected for full-text screening were divided, and each half was screened by two members of the review team to confirm their eligibility against the inclusion criteria. During screening, six studies in disagreement were discussed until consensus was reached. Of the six studies discussed, three were included within the final review. The other three studies were excluded due to no reporting of data on ethnicity (*n* = 1), based in primary care (*n* = 1) and being out of scope (*n* = 1).

### Data Extraction

Key data related to the population being studied, participants’ demographics, location and treatment settings were extracted from the eligible articles using a standard data extraction template [22]. A descriptive synthesis of the extracted data was then performed.

### Study Quality Appraisal

Given this is a scoping review, the traditional assessment of risk of bias was not appropriate as the purpose of this review was to draw broad conclusions of the cumulative evidence from heterogeneous sources [20].

## Results

**Fig. 1:**
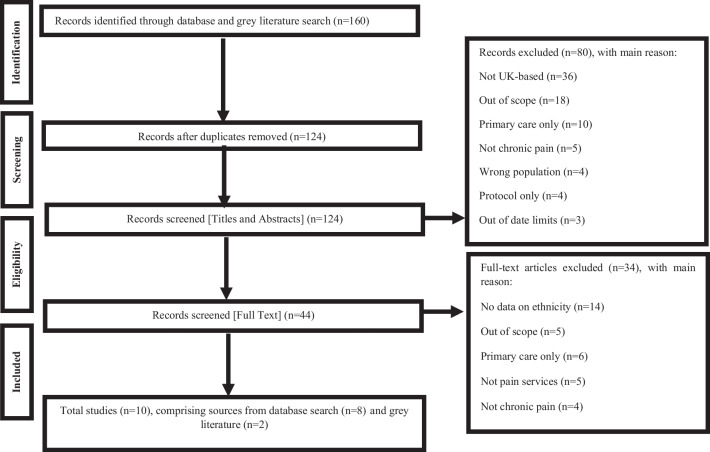
Flowchart summarising the review process and reasons for exclusion

The literature searches identified 124 records after duplicates were removed. Eighty records were excluded after the title and abstract screening. Forty-four full texts were assessed for eligibility. Thirty-four records were excluded after full-text screening. Figure 1 summarises the review process and reasons for exclusion. Ten articles met the inclusion criteria and comprised peer-reviewed articles (*n* = 3), poster abstracts (*n* = 5) and public reports (*n* = 2) (Table 2). Further characteristics are presented in Supplementary Material Table 3.

**Table 2:** Included articles

Author and DOI	Title	Study Details	Journal	Database from which retrieved
Baker et al. 2021 [23] 10.1080/09540121.2020.1869148	I have failed to separate my HIV from this pain: the challenge of managing chronic pain among people with HIV’	Peer-reviewed Full text paper Qualitative focus groups	AIDS Care	Embase
Bhatti-Ali et al. 2019 [24] 10.1177/204946371983653	Evaluation of a culturally adapted pain management programme	Poster abstract Quantitative study Pilot prospective study	British Journal of Pain	Embase
Burton et al. 2019 [25] 10.1002/msc.1400	Exploring thoughts about pain and pain management: Interviews with South Asian community members in the UK	Peer-reviewed Full text paper Qualitative interviews	Musculoskeletal Care	Embase
El-Damanawi et al. 2021 [26] 10.1093/ndt/gfaa139.SO090	Evaluating pain in autosomal dominant polycystic kidney disease	Full text paper Quantitative study Feasibility RCT study	Nephrology, Dialysis Transplantation	Embase
Garvey et al. 2014 [27] 10.1111/hiv.12147	Is appropriate evaluation of male subjects with chronic pelvic pain feasible within a specialist GU service?	Poster abstract Quantitative study Retrospective cohort study	HIV Medicine	Embase
Gauntlett-Gilbert et al. 2018 [28] 10.1177/2049463718762348	Across the lifespan: Chronic pain-related disability at different developmental stages	Poster abstract Quantitative study Retrospective cohort study	British Journal of Pain	Embase
Shoiab et al. 2016 [29] 10.1016/j.physio.2016.10.240	A language specific and culturally adapted pain management programme	Poster abstract Quantitative study Service evaluation	Physiotherapy	CINAHL
Park et al. 2021 [30] 10.1111/bjh.17492	The therapeutic mechanisms that are unique in a sickle cell pain management programme. A grounded theory	Poster abstract Qualitative interviews	British Journal of Haematology	Embase
British Pain Society 2013 [8] https://www.britishpainsociety.org/static/uploads/resources/files/members_articles_npa_2013_safety_outcomes.pdf	The National Pain Audit Third Report	Grey literature National audit	N/A	OpenGrey
Public Health England 2017 [5] https://assets.publishing.service.gov.uk/government/uploads/system/uploads/attachment_data/file/940858/Chronic_Pain_Report.pdf	Chronic pain in adults	Grey literature National survey	N/A	OpenGrey

### Study characteristics

All studies (*n* = 10) were published between 2013 and 2021. Study size ranged between six and 13,193 participants with most studies having a higher ratio of female to male participants. Half of the studies (*n* = 5) involved more participants in middle adulthood (between the ages of 35 and 65) (Supplementary Material Table 3). Five studies were published in international health journals [23, 25–27, 29]. Three studies were published in UK health journals [24, 28, 30]. No studies were published in health journals with a specific focus on race, ethnicity, or inequalities. Table 3 presents the characteristics of the included studies.

**Table 3:** Characteristics of studies included in the scoping review

Study characteristic		Number of studies
Geographical region of the UK	UK-wide	1
	England and Wales	1
	England only	8
Chronic pain service	All provisions of care	4
	Secondary and Tertiary	6
Painful condition being studied/site of chronic pain	**Generalised chronic pain**	**6**
	**Specific pain condition**:	**4**
	- HIV-related chronic pain	1
	- Autosomal dominant polycystic kidney disease–related chronic pain	1
	- Sickle cell disease–related chronic pain	1
	- Male pelvic chronic pain	1
Study design	**Qualitative:**	**3**
	- Focus groups	1
	- Interviews	2
	**Quantitative:**	**5**
	- Feasibility RCT	1
	- Pilot prospective	1
	- Retrospective cohort	2
	- Service evaluation	1
	**Grey literature:**	**2**
	- National audit	1
	- National report	1

Four studies involved participants from urban areas of England including Birmingham, London, Walsall, Stafford, Barnsley and Bradford [25, 27, 29, 30]. Two studies recruited participants from rural areas including Cambridgeshire and Buckinghamshire [24, 26]. Three studies recruited participants from across the UK and so may have recruited participants from both rural and urban area, but this was not reported [5, 8, 28].

Eight studies assessed chronic pain across the entire body [5, 8, 23–25, 28–30], two at a particular anatomical site [26, 27] and four studied adults with conditions associated with chronic pain including HIV and sickle cell disease [23, 26, 27, 30].

Four studies included patients from primary care as well as secondary and tertiary care within their research [5, 23, 25, 26].

### Minority ethnic groups reported in studies

The breakdown of recorded ethnicities for each included study from the bibliographic database search (*n* = 8) is presented in Table 4.

**Table 4:** Ethnicity breakdown by study (not including grey literature sources)

Author	Sample size of entire study	Ethnicity breakdown of sample (%)					
		White British groups	Black ethnic groups	Asian ethnic groups	Other ethnic groups	Ethnicity kept confidential	Ethnicity of remaining sample not stated
Baker et al. 2021 [23]	39	20.5	79.5	-	-	-	-
Bhatti-Ali et al. 2019 [24]	7	-	-	100	-	-	-
Burton et al. 2019 [25]	10	-	-	100	-	-	-
El-Damanawi et al. 2021 [26]	42	90	-	-	-	-	10
Garvey et al. 2014 [27]	53	42	-	42	16	-	-
Gauntlett-Gilbert et al. 2018 [28]	805	96	-	-	-	-	4
Shoiab et al. 2016 [29]	6	-	-	100	-	-	-
Park et al. 2021 [30]	12	-	50	-	33	17	-

Three studies explored ethnicity as a primary research objective [23, 24, 29], and all of these studies considered South Asian individuals as one specific minority ethnic group.

Five studies reported the ethnic breakdown of all participants [5, 8, 23, 27, 30]. Two studies recorded the number of white British participants only, constituting 96–98% [28] and 90% [26] of their samples, respectively. However, these studies did not categorise any other ethnic groups in their remaining sample (2% [28] and 10% [26]). None of the reviewed studies specifically focused on black ethnicities, with only four studies reporting data for participants from black ethnic groups [5, 8, 23, 30]. Five studies had at least one participant from an Asian ethnic group [5, 24, 25, 27, 29]. No study reported participants with mixed ethnicity.

All ten studies presented some categorisation of ethnicity data for those with chronic pain. Garvey et al. (2014) used ‘other’ as a catch-all descriptor for ethnicity other than white British or British Asian [27]. Five studies listed the ethnic groups of all participants [23–25, 29, 30]. These five studies included more participants from minority ethnic groups than white British participants [23–25, 29, 30].

Only one study [30] that did not restrict their participants to one specific ethnic group, did not include any white British participants.

## Discussion

### Overview

This study set out to gain initial data on the extent to which minority ethnic groups with chronic pain access secondary and tertiary chronic pain services in the UK. In our review, only 10 out of the 124 records met the basic requirements of the inclusion criteria, namely, stating the ethnicities of the adult sample and UK chronic pain services. A combination of full-text papers and abstracts were reviewed which highlights the lack of published data relating to minority ethnic groups access to UK chronic pain services. Overall, we found a paucity of published data related to access to UK chronic pain services for adults from minority ethnic groups.

Some studies only recorded ‘white British’ and no other ethnicities which may suggest a lack of accurate collection about non-white British ethnic groups that may be attending UK chronic pain services, or it may be that minority ethnic groups were not included in the research. We will discuss possible reasons for this further below.

### Ethnicity breakdown of chronic pain service populations

Due to the limited data obtained from our review, we cannot draw conclusions on the access to chronic pain services for individual minority ethnic groups. Our review found no studies which specifically attempted to determine the ethnic profile of pain service populations. There were also no studies which specifically assessed ethnic disparities within chronic pain services. The third national pain audit highlighted a concern that access to specialist chronic pain services may be inconsistent and difficult for minority ethnic groups compared to those who are white British [8]. Given the greater incidence of chronic pain within some minority ethnic groups in the UK [4], the known disparities in chronic pain treatments between ethnic groups [14–16] and the limited published evidence on minority ethnic groups attending chronic pain services highlighted by this scoping review, there may be reason to suggest disparities in accessing chronic pain services for minority ethnic groups. However, until accurate ethnicity records are routinely collected in UK chronic pain services, it is not known how many adults from minority ethnic groups are attending these services or whether there is disparity in access. This highlights the need for more research to address access to chronic pain services in the UK for adults from minority ethnic groups.

Evidence suggests self-reported chronic pain is more prevalent amongst minority ethnic groups compared to white ethnic groups in the UK [32–34]. Within the literature, the use of out-patient chronic pain services has been noted to be significantly lower amongst minority ethnic groups than white British in the UK [35, 36]. If these assumptions are true and access to chronic pain services were equitable between different ethnic groups, a larger proportion of minority ethnic groups accessing chronic pain services would be reported (as compared to the proportion of minority ethnic groups in the UK population). However, our scoping review of the available literature is unable to provide meaningful comment on this observation given that the main finding from this review highlights underrepresentation of minority ethnic groups in chronic pain research as opposed to inequitable access. However, the largest UK-based quantitative study (*n* = 805) within this review reported that 96% of participants were white British [28]. This is a higher proportion than one would expect considering that according to the 2021 UK census, 74.4% of the UK population identified themselves as white English, Welsh, Scottish, Northern Irish or British [11] which one could speculate means poorer access to chronic pain services for adults from minority ethnic groups. Therefore, further research is required to test this hypothesis.

There have been calls from charitable organisations and government to address unfair differences in accessing services for chronic conditions such as diabetes [37] [38]. However, no such national calls have been made regarding access to chronic pain services for minority ethnic groups despite the British national pain audit highlighting that access to specialist chronic pain services may be inconsistent and difficult for minority ethnic groups compared to those who are white British [8]. Our scoping review finds little published evidence relating to minority ethnic groups accessing chronic pain services for specific chronic conditions. Only four of the ten studies studied adults with chronic conditions associated with chronic pain namely HIV, kidney disease and sickle cell disease [23, 26, 27, 30].

None of the reviewed studies specifically focused on black ethnic groups and only four out of the ten reviewed studies had one or more participants from a black ethnic group. The two studies that involved more participants from minority ethnic groups compared to white British assessed participants with the long-term conditions HIV and sickle cell disease [23, 30]. These conditions are known to have a higher prevalence in minority ethnic groups, specifically adults from black African and Afro-Caribbean backgrounds [39, 40].

The literature suggests adults of Asian ethnic groups are more willing to self-report pain but attend specialist treatment settings in secondary and tertiary care less readily [3, 4]. Our review found only three studies assessing access to chronic pain services by South Asian ethnic groups in the UK [24, 25, 29]. All three studies concluded that delivering a culturally adapted chronic pain programme would enable successful engagement of people from Asian ethnic groups to chronic pain services [24, 25, 29]. The best way to acknowledge cultural differences in the perceptions and experiences of chronic pain and tailor the service provided accordingly to address these differences is unclear. Therefore, more research on culturally adapted chronic pain services and whether this would improve access to chronic pain services by Asian ethnic groups is needed.

### Underrepresentation of minority ethnic groups in other healthcare research

This study draws a link to the persistent issues in the social construction of race and tangible inequalities in the healthcare system which is well documented in the literature [41]. The limited representation of ethnicity within our findings reflect that found in other areas of healthcare research including the COVID-19 pandemic [42–47]. For example, a systematic review investigating the impact of ethnicity on clinical outcomes in COVID-19 revealed that only 5 out of 207 articles reported ethnicities of participants [47]. Reasons for this lack of documentation are unclear, despite general agreement that ethnicity data collection is essential to better understand and reduce disparities in healthcare given it has been suggested structural racism is a potential public health risk within the UK [48]. The authors speculate this may be due to minority ethnic groups not being adequately represented within chronic pain research and/or reflect a bias in those who conduct such research to not adequately engage with these populations. This is despite studies in other areas of healthcare research, such as cardiovascular disease [49] and gerontology [50], highlighting that minority ethnic groups are willing to participate in research if the study has direct relevance to them and their communities and if they are approached with sensitivity and given clear explanations of what participation involves [43]. Increasing efforts to engage with minority ethnic groups in order to encourage participation in chronic pain research are required. Some studies have highlighted that inadequate resources for aspects such as translation services, communication and socio-cultural factors, stigma and inadequate finances to attend treatment are potential reasons why adults from minority ethnic groups are not represented in healthcare research [48, 50–52].

Within the UK, minority ethnic groups generally have worse health than the overall population [53] with chronic pain disproportionately affecting some minority ethnic groups [4]. NHS England recognises race and ethnicity as drivers of healthcare inequalities [13]. To reduce racial and ethnic health inequalities within UK chronic pain services, the construct of racial and ethnic consciousness needs to be considered. This discussion is well-established within the United States, Australia and New Zealand, and it may influence the decisions of adults from minority ethnic groups regarding their health needs (including accessing chronic pain services or participating in chronic pain research) [54, 55].

### Inconsistencies in terminology describing ethnicity

The terminology used to describe ethnicity and how ethnicity data is recorded varied across the reviewed studies which is consistent with supporting literature assessing ethnicity documentation within healthcare [46, 47]. The inconsistent documentation of ethnicity across the reviewed studies may be attributable to the fact that conversations around ethnicity and race can make people feel uncomfortable [56]. Furthermore, the way people may describe their ethnicity varies as ethnicity is unique to each individual [57]. Perhaps, some people may consider ethnicity to be personal so are unwilling to divulge this information with chronic pain services. The fact that no data regarding participants from mixed ethnic backgrounds were identified in the reviewed studies further highlights an absence of information within ethnicity data collection in chronic pain services and research.

Consensus needs to be reached regarding accurate groupings of ethnicities, including those from mixed ethnic backgrounds. This is difficult and made more so by the fact that two individuals from the same ethnic background may categorise themselves in different ways. Using the ethnicity categories from the 2021 UK census may be a viable option; however, there is huge diversity within broader ethnic groups. We acknowledge that ethnicity categories are socially constructed, and ethnic diversity varies across the geography of the UK. Therefore, establishing a list of consistent ethnic categories to be used across all chronic pain services in the UK may be difficult. However, once ethnicities are defined and recorded more accurately, we can better establish the degree to which there are healthcare disparities in the access to chronic pain services. This can facilitate the barriers to accessing chronic pain services for minority ethnic groups to be identified and addressed in future research.

### Strengths and limitations

Ethnic disparities in attending chronic pain services have been assessed in countries outside of Europe including New Zealand [58] and America [59]. The focus of this review are chronic pain services in the UK, so the findings are not generalisable or comparable to other countries or healthcare settings. Our findings are novel as there are no comparable published UK-based or European reviews assessing access to chronic pain services for adults from minority ethnic groups. However, it would be interesting to observe whether results are similar elsewhere in the European Union, especially in locations with different ethnic population sizes to the UK. The findings are applicable to the adult population only. The paediatric population were not in the scope of this review. The review excluded non-English language papers as we were specifically focusing on UK chronic pain services. In consultation with academic librarians, the authors omitted broader search terms including *pain from the search strategy in line with our published protocol given that the focus of the study was chronic pain and chronic pain services [22]. However, we acknowledge that including this wildcard term within the search strategy may have increased the number of search results.

Broad input and consultation from clinical experts, experienced researchers, an epidemiologist with expertise in chronic pain, academic librarians and people from minority ethnic groups was sought in designing a rigorous approach to this review. Unpublished articles and grey literature were included; thus, this review is comprehensive, and the risk of publication bias has been minimised. However, we acknowledge that our scientific database search did not include non-published NHS chronic pain service clinical data which may have provided useful ethnicity information.

## Conclusion

To our knowledge, this is the first review to assess the current status of adults from minority ethnic groups accessing chronic pain services in secondary and/or tertiary care in the UK. We found little published ethnic information suggesting that accurate categorisation and recording of ethnicities and their representation in UK chronic pain services is limited. The need for further research related to accessing chronic pain services in secondary and/or tertiary care in the UK is highlighted. More robust data on this topic is needed as it appears chronic pain services in the UK are not routinely collecting ethnicity data and increased involvement of minority ethnic groups within chronic pain research is needed. Findings from this review will inform future research regarding barriers and facilitators to accessing secondary and/or tertiary chronic pain services in the UK for adults from minority ethnic groups with chronic pain. This future work is essential in identifying healthcare disparity issues for adults from minority ethnic groups that can be addressed by chronic pain services to remove such inequalities.

## Supplementary information

ESM 1 (DOCX 29 kb)

## Data Availability

A data availability statement is not required as no new data were created or analysed in this study.
