# Supplementary material for: Access to Chronic Pain Services for Adults from Minority Ethnic Groups in the United Kingdom (UK): a Scoping Review
Source: J Racial Ethn Health Disparities. 2023 Oct 16;11(6):3498–508. doi: 10.1007/s40615-023-01803-2 (PMC11564250; doi:10.1007/s40615-023-01803-2)
Supplement: Supplementary file 1 — (DOCX 29 kb) [file 40615_2023_1803_MOESM1_ESM.docx]

**Supplementary Material Tables**

**Access to chronic pain services for adults from Minority Ethnic groups in the United Kingdom (UK): A scoping review.**

Journal of Racial and Ethnic Health Disparities

Authors: Emily Leach^1,2^, Mwidimi Ndosi^1^, Gareth Jones^3^, Helen Ambler^4^, Sophie Park^4^, Jennifer S. Lewis^1,4^

Affiliations:

1. School of Health and Social Wellbeing, University of the West of England, Bristol, United Kingdom
2. Solent NHS Podiatry, Solent NHS Trust, Southampton, United Kingdom
3. School of Medicine, Medical Sciences and Nutrition, University of Aberdeen, Aberdeen, United Kingdom
4. Bath Centre for Pain Services, Royal United Hospitals Bath NHS Trust, Bristol, United Kingdom

Email address of corresponding author Emily Leach: Emily.Leach@solent.nhs.uk

Supplementary Material Table 1: PICO MODEL

| PICO Element | Description |
| --- | --- |
| Population | Adults in the UK with chronic pain or a condition associated with chronic pain [from Minority Ethnic groups]. |
| Intervention | Access to [regional/national] chronic pain services [in secondary and tertiary care] within the UK. |
| Comparison | Comparing this to what would be expected given the ethnic breakdown of the UK population and chronic pain incidence within the UK population and different ethnic groups. |
| Outcome | Number of adults in the UK with chronic pain from Minority Ethnic groups who are accessing chronic pain services – are they represented? |

Supplementary Material Table 2: Search Strategy

| 1 | “chronic pain” or “long-term pain” or “long term pain” or “persistent pain” |
| --- | --- |
|  | AND |
| 2 | “pain service” or “pain treatment” or “pain management” or “pain program*” or “pain management program*” or “pain clinic” or “pain centre” or “pain center*” or “secondary care” or “tertiary care” or “multidisciplinary team” or “multidisciplinary approach” or “multidisciplinary care” or “multidisciplinary management” or “multidisciplinary treatment*” or “interdisciplinary approach” or “interdisciplinary team” or “interdisciplinary care” or “interdisciplinary treatment*” or “collaborative care” or “MDT” |
|  | AND |
| 3a | “UK” or “United Kingdom” or “Great Britain” or “GB” or “British” or “national health service” or “NHS” or “England” or “Scotland” or “Wales” or “Northern Ireland” or “Scottish” or “Welsh” or or “Northern Irish” or “English” |
|  | NOT |
| 3b | “British Columbia” or “New England” or “New South Wales” or “United States” |
|  | AND |
| 4a | “BAME” or “BME” or “ethnic disparit*” or “Ethnic*” or “ethnic minorit*” or “ethnic difference*” or “ethnic group*” or “minority group*”or “mixed ethnic*” or “multiple ethnic*” or “black*” or “black people*” or “black person*” or “Asian*” or “British Asian*” or “Jew*” or “Irish traveller*” or “traveller*” or “gyps*” or “roma gyps*” or “romany gyps*” or “white*” or “white people*” or “white person*” or “white other” or “white mixed” or “Caucasian” or “eastern European” or “white British” or “people of colour” or “mixed race” or “immigrant*” or “migrant*” or “asylum seeker*” or “refugee” or “race” or “race factor” or “race difference*” |
|  | NOT |
| 4b | “White” AU |
|  |  |
|  | Narrow by English Language and Publication Date [2004-2021] |

Supplementary Material Table 3: Data Extraction Table

| Author | Study design | Geographical location | Setting | Description of chronic pain / condition associated with chronic pain | Participant details | Ethnicity details | Sample size |
| --- | --- | --- | --- | --- | --- | --- | --- |
| Baker et al. 2021 [31]  “I have failed to separate my HIV from this pain: the challenge of managing chronic pain among people with HIV.”  Full text paper | Qualitative  Focus groups | UK-wide | All Levels of Care  Participants recruited from the UK Community Advisory Board (UK-CAB)’s online forum and HIV advocacy  organisations affiliated with the UK-CAB. | Painful condition: HIV  Chronic pain as defined by the International Association for the Study of Pain (i.e., living with ongoing pain for more than three months) | Persons with HIV aged at least 18 years. (n=39)  Age breakdown: 35-44 (n=3) (7.7%), 45-54 (n=16) (41.0%), 55-64 (n=14) (35.9%), 65-75 (n=4) (10.3%), prefer not to say (n=2) (5.1%)  Gender breakdown: male n=15 (38.5%), female (n=24) (61.5%)  Financial situation breakdown: comfortable (n=2) (5.1%), coping n=9 (23.15%), difficult (n=14) (35.9%), very difficult (n=10) (25.6%), prefer not to say (n=4) (10.3%).  Education breakdown: primary (n=1) (2.6%), secondary (n=8) (20.5%), post secondary n=27 (69.2%), prefer not to say (n=3), missing (n=2)  Sexual orientation heterosexual (n=31) (79.5%), homosexual (n=6) (15.4%), prefer not to say (n=2) (5.1%) | Black African (n=28) (71.8%), Black Caribbean (n=3) (7.7%), White (n=8) (20.5%) | (n=39)  Purposive sampling |
| Bhatti-Ali et al. 2019 [29]  “Evaluation of a culturally adapted pain management programme”  Abstract only | Quantitative study  Pilot prospective study | England: Barnsley  INHEALTH pain management service | Secondary care,  Multidisciplinary pain management programme involving physiotherapist and 2 psychologists. | Generalised pain including chronic pain, no definition given | Urdu speaking individuals referred to the specialist INHEALTH pain management service (n=7)  Male (n=2), Female (n=5) | Urdu speaking individuals (n=7) 100% of sample | (n=7)  Sampling strategy not specified |
| Burton et al. 2019 [24]  “Exploring thoughts about pain and pain management: Interviews with South Asian community members in the UK”  Full text paper | Qualitative study  Interviews | England: Birmingham, Buckinghamshire, London, Walsall, Stafford | Not specified, assessing pain management programmes which may involve all provisions of care | Chronic pain defined by the British Pain Society 2013 as “common complex sensory, emotional, cognitive and behavioural long‐term health condition which occurs when pain cannot be resolved by available medical or other treatments”  However, only one of the 10 participants had chronic pain. | South Asian adults (n=10)  Age range = 19-46.  Gender breakdown: female (n=7), male (n=3)  Religion Breakdown: Sikh (n=6), Hindu (n=3), Christian (n=1);  Location breakdown: London (n=3), Birmingham (n=3), Buckinghamshire (n=1), Walsall (n=1), Stafford (n=2) | South Asian (n= 10) (100%) | 10  Snowball sampling.  Project information was circulated to community‐based groups and Mosques in Birmingham, and placed as advertisements on social media, online discussion boards and mailing lists. |
| El-Damanawi et al. 2021 [28]  “Evaluating pain in autosomal dominant polycystic kidney disease”  Full text paper | Quantitative study  Feasibility RCT study | England: Cambridgeshire | Not specified, study included pain specialists (secondary care) | Pain condition: Autosomal Dominant Polycystic Kidney Disease-related chronic pain.  Classifies pain into categories: no pain, mild pain, moderate pain and severe pain. | Adult ADPKD patients with an eGFR >20mls/min/1.73m2 (n=42)  mean age of respondents was 47 +-13 years, 59%  Female (n=23), Male (n=19)  median disease duration was 14.2 years  69% had enlarged kidneys, 64% had hypertension, hepatic cysts present in 59% | 90% (n=35) were White British | (n=42) recruited to trial however (n=39) completed  Sampling strategy not specified |
| Garvey et al. 2014 [25]  “Is appropriate evaluation of male subjects with chronic pelvic pain feasible within a specialist GU service?”  Abstract only | Quantitative study  Retrospective cohort study | England: London | Secondary care,  Male Problem Clinic at the Jefferiss Wing, St Mary’s Hospital | Male chronic pelvic pain, no definition given | New patients referred to male problem clinic (n=53)  Mean age 39.3  100% of sample male (n=53)  Most patients (94%) reported chronic pain (mostly penile, perineal and scrotal), plus urethral symptoms and/or voiding symptoms in 17 and 20% respectively.  Symptoms had been present for an average of 21 months. | White British (42%), British Asian (42%), other (16%) | (n=53)  No sample strategy specified |
| Gauntlett-Gilbert et al. 2018 [30]  “Across the lifespan: Chronic pain-related disability at different developmental stages”  Abstract only | Quantitative study  Retrospective cohort study | UK-wide | Tertiary Care residential or inpatient specialist pain rehabilitation programme at the Bath Centre for Pain Services | Chronic pain, no definition given | (n=805) adults who attended a residential or inpatient specialist pain rehabilitation programme  25yrs< (n=119), 20.9yrs, 87% female, chronicity 60 months;  aged 26-54 (n=545), 41.5 yrs, 65% female, chronicity 85 months;  aged 55> (n=141), 60.1yrs, 64% female, chronicity 170months | 25yrs<: 98% White British  26-54: 96% White British  Aged >55yrs: 96% White British | (n=805)  Sample strategy not specified |
| Shoiab et al. 2016 [27]  “A language specific and culturally adapted pain management programme”  Abstract only | Quantitative study  Service evaluation | England: Bradford | Secondary/Tertiary Care - 'Living with pain team' MDT PMP consisting of a Physiotherapist, Clinical Psychologist, GP with Special Interest in Pain Management | Generalised pain, no definition given | 6 Urdu speaking individuals.  Female (n=5), Male (=1) | Urdu-speaking individuals (n=6) 100% of sample | (n=6), sampling strategy not specified |
| British Pain Society 2013 [8]  “The National Pain Audit Third Report”  Full report | Grey literature  National audit | UK: England and Wales | Secondary and tertiary care – pain management services: These varied hugely from subspecialty clinics within a single hospital to county-wide services over a large geographical area. Pain clinic (n=27), MDT pain clinic (n=111), MDT pain centre (n=34) | All types of pain (77% had spinal pain including low back, neck pain and sciatica. 17% had neuropathic pain only.)  Chronic pain as defined by the International Association for the Study of Pain (i.e., living with ongoing pain for more than three months) | (n=1,799) patients who responded to questionnaire  The average age of the 12-month responders was 59 years (standard deviation 15); range from 6 to 96 years,  Female (65%) Male (35%)  70% had been referred by their  GPs. | Ethnicity was collected for the first time in this audit round. The overwhelming ethnicity of respondents was white, reported at 92.9%. | (n=1,799), no sample strategy specified |
| Public Health England 2017 [5]  “Chronic pain in adults”  Full report | Grey literature  National report | England | All provisions of care, mentions ‘specialist pain service’ | All types of pain, used their own definition | (n=7,997) adults interviewed, (n=5,196) visited by nurse  The prevalence of chronic pain increased with age ranging from 18% among those aged 16-34 years to 53% among those 75 years and over.  Age 45 to 54 years (39%) was the point when chronic pain became significantly higher than the average for all adults.  Women (38%) reported a higher prevalence of chronic pain than men (30%). Women reported higher chronic pain prevalence at every age group than men. Prevalence of chronic pain increased with age for both men and women. | All ethnic groups showed similar reporting of chronic pain to the figure for all persons of 34%, except for people in the Black ethnic group who reported a significantly higher prevalence at 44%.  The percentage of people with musculoskeletal, heart and circulatory system and/or mental health disorders that reported chronic pain were similar between White, Asian and Black groups. However, among those reporting no long-lasting illness, prevalence of chronic pain was higher amongst the Asian (43%) group than the White group (33%). Smaller sample size than the Asian group and therefore has a wider confidence interval  Among those with chronic pain, people in the Asian ethnic group (42%) were more likely to report pain that had a high interference on their usual activities than the White group. | (n=7,997) adults interviewed, (n=5,196) visited by nurse, no sampling strategy specified |
| Park et al. 2020 [26]  “The therapeutic mechanisms that are unique in a sickle cell pain management programme. A grounded theory”  Abstract only | Qualitative study  Interviews | UK: Metropolitan city in the UK, not specified | Tertiary specialist pain service: 8 participants who attended SPMP at General pain services and 2 from haematology services | Painful condition: Pain associated with sickle cell disease.  Discussed both acute and chronic pain. Chronic pain defined by Elliot et al. 1999 as “pain or discomfort that is persistent or sporadic, can last for more than three months”. | (n=8) participants from general pain services and (n=2) from haematology service  Age range of participants: 35-58  Female (n=10) (100%)  Employment status: Self-employed (n=3), student (n=1), Homemaker/carer (n=2), part-time (n=1), full-time (n=1), retired (n=1), unemployed (n=1)  Time since attended PMP:  >1Year = 100%  Current wellbeing: poor (n=2), either good or poor(n=4), good (n=4) and PMP helpfulness: helpful (n=4), very helpful (n=8) | Black African (n=3) Black British (n=3) British African (n=3) African (n-1) Kept confidential (n=2) | (n=12)  3 stage recruitment  8 (initially 9 but 1 unable to take part due to health reasons) who attend SPMP in metropolitan city contacted. Stage 2 = 2 facilitators (physiotherapist and psychologist) of the same SPMP  Stage 3 = 2 participants recruited from general PMP in haematology service of another NHS Trust |

Key:

Full Text Paper

Abstract Only
